# Feasibility study of finalizing the extended adjuvant temozolomide based on methionine positron emission tomography (Met-PET) findings in patients with glioblastoma

**DOI:** 10.1038/s41598-019-54398-2

**Published:** 2019-11-28

**Authors:** Seiichiro Hirono, Yuzo Hasegawa, Tsukasa Sakaida, Yoshio Uchino, Kazuo Hatano, Toshihiko Iuchi

**Affiliations:** 1 Division of Neurological Surgery, Chiba Cancer Center, 666-2 Nitona-cho, Chuo-ku, Chiba 260-8717 Japan; 2 Division of Nuclear Medicine Chiba Ryogo Center, 3-30-1 Isobe, Mihama-ku, Chiba 261-0012 Japan; 3 Division of Radiation Oncology, Tokyo Bay Advanced Imaging and Radiation Oncology Clinic, 1-17 Toyosuna, Mihama-ku, Chiba 261-0024 Japan

**Keywords:** CNS cancer, Surgical oncology

## Abstract

In the management of patients with newly diagnosed glioblastoma, there is no standard duration for adjuvant temozolomide treatment. This study aimed to assess the feasibility of finalizing adjuvant temozolomide treatment on the basis of methionine uptake in methionine positron emission tomography (Met-PET). We conducted a retrospective review of glioblastoma patients who underwent more than twelve cycles of temozolomide (extended temozolomide) treatment after resection and concomitant chemoradiotherapy with no evidence of recurrence on MRI. In addition to the methionine uptake value at the completion of extended temozolomide, local and distant recurrence and progression-free survival were also analyzed. Forty-four patients completed the extended temozolomide treatment. Among these, 18 experienced some type of tumor recurrence within one year. A Tmax/Nave value of 2.0 was the optimal cut-off value indicating progression. More than 80% of the patients with low methionine uptake completed the temozolomide treatment, and subsequent basic MRI observations showed no recurrence within one year after Met-PET. Subgroups with high uptake (≥2.0), even with continuation of temozolomide treatment, showed more frequent tumor progression than patients with low uptake (<2.0) who completed the extended temozolomide treatment (p < 0.001, odds ratio 14.7, 95% CI 3.46–62.3). The tumor recurrence rate increased in stepwise manner according to methionine uptake. Finalization of the extended temozolomide treatment on the basis of low uptake value was feasible with a low recurrence rate. Compared to MRI, Met-PET shows better ability to predict tumor progression in long-term glioblastoma survivors with extended temozolomide use.

## Introduction

Glioblastoma multiform (GBM) is the most malignant and aggressive neoplasm occurring in the central nervous system. Currently, the standard of care for newly diagnosed GBM consists of a combination of maximal safe resection with concurrent temozolomide (TMZ) and radiotherapy followed by adjuvant TMZ (five days per month), the Stupp protocol, which yields a median overall survival (OS) ranging from 12.7 to 21.7 months according to the methylation status of O^6^-methylguanin-DNA methyltransferase (MGMT) gene^1^.

One of the problems in clinical care in this regimen is that there is no standard period for adjuvant TMZ treatment following concurrent chemoradiotherapy. The majority of recent clinical trials used up to six or twelve cycles of adjuvant TMZ treatment for 5 days of every 28-day cycle after initial chemoradiotherapy^1–7^. TMZ is an oral alkylating agent that induces apoptosis in glioma cells with a feasible tolerability and acceptable toxicity. Long-term adjuvant TMZ treatment with a mean of 27 cycles has been reported to be feasible and safe^8^. Additionally, Seiz *et al*. demonstrated the positive correlation between progression-free survival (PFS) and OS and the number of TMZ treatment cycles in GBM patients treated with long-term TMZ administration (ranging from 6 to 57 cycles)^9^. More specifically, Blumenthal showed that prolonged TMZ use led to prolonged PFS only in patients with methylated MGMT genes^10^. However, severe side effects of TMZ treatment, including thrombocytopenia, neutropenia, and opportunistic infections including *Pneumocystis carinii*^1,2^, are not rare, and are sometimes lethal. Moreover, the risk of secondary carcinogenesis, impaired fertility, and mutagenesis in the retinoblastoma (Rb) and mammalian target of rapamycin (AKT-mTOR) pathways^11^ has also been reported. These findings raise questions about patient eligibility for prolonged adjuvant TMZ therapy, and the patient characteristics associated with treatment finalization.

Metabolic imaging with positron emission tomography (PET) has been applied to many clinical settings, including glioma management, and this approach offers the advantage of allowing visualization of tumor metabolic activity. Methionine is one of essential amino acids that diffuse into the cell via the amino acid transport system or the blood brain barrier^12^, and it has an important role in protein metabolism. The successful use of ^11^C-Methionine PET (Met-PET) has been recently reported in glioma diagnosis^13^, recurrence and radiation necrosis^14^, and surgical planning^15^. In addition, we have also reported the feasibility of radiation therapy planning for GBM based on the findings of Met-PET prior to radiation therapy^16^. In these circumstances, this approach may also be helpful to select the possible candidates for finalization of prolonged adjuvant TMZ therapy and simple follow-up management among long-term GBM survivors.

In this retrospective case–control study, the authors aimed to clarify the possible predictive role of Met-PET, especially in adjuvant TMZ treatment finalization or continuation, among GBM patients who had received more than 12 cycles of adjuvant TMZ treatment with no evidence of tumor recurrence on routine MRI surveillance.

## Methods

### Patient inclusion criteria

Patients with newly diagnosed and pathologically confirmed GBM who had undergone standard postoperative therapy^2^ that consisted of radiotherapy plus concomitant daily TMZ treatment, followed by adjuvant TMZ treatment were identified in our hospital records retrospectively. Concomitant TMZ treatment consisted of oral TMZ at a daily dose of 75 mg/m^2^ given 7 days per week from the first to the last day of radiotherapy, for a maximum of 49 days. After a 4-week break, patients received at least 12 cycles of adjuvant TMZ treatment (150–200 mg/m^2^) for 5 days every 28 days until tumor recurrence, progression, or intolerable toxicity. MRI with contrast enhancement at SIEMENS MAGNETOM 1.5 tesla scanner (TR 10 msec, TE 3.5 s, flip angle 20°, field of view 240 mm, slice thickness 1.8 mm, no interslice gap, and matrix 320 × 320) or 3.0 tesla scanner (TR 10 msec, TE 2.95 s, flip angle 18°, field of view 240 mm, slice thickness 1.8 mm, no interslice gap, and matrix 384 × 384) was repeated in all patients every 1 or 2 months after the surgery as a routine clinical evaluation. Met-PET was also performed at the doctor’s discretion as a part of clinical evaluation. Patients who satisfied the following four criteria were enrolled into this retrospective analysis; (1) newly diagnosed and pathologically confirmed GBM, (2) completed the concomitant TMZ treatment and at least twelve cycles of adjuvant TMZ (extended TMZ) treatment, (3) no evidence of recurrence or progression on MRI at the completion of the 12th cycle of adjuvant TMZ treatment, 4) underwent Met-PET scans following the 12th or more cycle of adjuvant TMZ treatment.

Among the 226 GBM patients who were treated with TMZ at our institution from 2006 to 2017, a total of 44 GBM patients were eligible and enrolled into this retrospective analysis. A total of 182 patients were excluded from the study owing to the following reasons: 49 patients who had not completed at least 12 cycles of adjuvant TMZ treatment were excluded, as well as 22 patients who withdrew from adjuvant TMZ treatment due to side effects, 45 patients who stopped the TMZ treatment due to deterioration in performance status, 48 who stopped the treatment due to tumor progression, and 18 additional patients with other reasons. The institutional ethical review board of Chiba Cancer Center approved this retrospective study (No.1742, December 2015) and waived the need for informed consent for our retrospective study. The study was complied with all tenets of the Declaration of Helsinki.

### Surgery and MGMT status

Surgical removal of the tumor prior to the radiotherapy plus concomitant daily TMZ treatment was performed in all cases. The extent of resection was calculated by volumetric evaluation of the enhancing tumor on MR images taken before surgery and within 72 hours after surgery. Methylation of the *O-6-*methylguanine-DNA methyltransferase (MGMT) gene promoter was evaluated using methylation-specific polymerase chain reaction.

### Met-PET procedure and evaluation of the uptake value

Met-PET scanning was performed using high-resolution fullring scanners (Discovery ST-E; GE Healthcare UK Ltd, Buchinghamshire, UK) with a spatial resolution of 4.8 mm at one of the affiliated hospitals. The patients fasted for ≥4 hours before PET scanning, and PET images were acquired with the patient in a resting state. Static scanning was performed for 6 minutes in 3-dimensional acquisition, and attenuation-corrected PET images were reconstructed using computed tomography (CT) data by means of a 3D-ordered subset expectation maximization algorithm (20 subsets and two iterations). A Met dose of 370–720 MBq was injected intravenously within 1 minute, with the scan starting 20 minutes after Met injection. Summation images covering 20 to 40 min after the injection were used for analysis. Met uptake was semiquantitatively evaluated using the ratio (Tmax/Nave) between the maximum uptake in the lesion to the mean uptake of 3 to 5 round regions of interest (ROIs) with a diameter of 1 cm drawn in the gray matter of the contralateral frontal or temporal lobe, avoiding the region affected by the tumor.

### Patient follow-up and recurrence evaluation

Routine clinical evaluation with contrast-enhanced MRI was performed in all patients at intervals of 1 or 2 months after the surgery. Met-PET was also performed at the doctor’s discretion. Recurrence or tumor progression were evaluated with the RANO criteria^17^. Local recurrence (LR) was defined as tumor progression around the original site of the tumor. Distant recurrence (DR) was defined as tumor progression at a noncontiguous region from the site where the tumor initially existed.

### Statistical analysis

The PFS at one year from the date of Met-PET was calculated using the Kaplan–Meier method. PFS was compared with the methionine uptake (Met-uptake) using logistic analysis to elucidate the Met-uptake value. The receiver operator characteristic (ROC) curve was drawn to determine the predictive value of Met-uptake following at least twelve cycles of adjuvant TMZ (extended TMZ) treatment, and the optimal threshold was calculated from this curve. The optimal cut-off point was the point on the ROC curve closest to the (0, 1) point. The areas under the ROC curves (AUC) were also calculated. Next, the Met-uptake was classified as a categorical variable using this optimal threshold, and the odds ratio (OR) was calculated to estimate the safety of completion of TMZ treatment based upon the results of Met-PET using logistic regression analysis. All statistical analyses were performed using JMP 11.2.1 software (SAS Institute, Cary, NC).

## Results

### Patients

Forty-four patients were enrolled in this study. The clinical and pathological characteristics of these patients are summarized in Table 1. Among these 44 patients, tumor recurrence was observed in 18 patients (41%) until 12 months after Met-PET. The recurrence pattern was LR for 11 lesions and DR for eight lesions. Subdural and cerebrospinal fluid dissemination were also observed in one patient each.

**Table 1:** Clinical characteristic of 44 patients.

Factor	Total	Low uptake group	High uptake group	p-value
No. of patients Male/female ratio	44 25:19	25 17:8	19 9:10	0.17
Median Tmax/Nave (range)	1.85 (1.00–3.50)	1.56 (1.00–1.93)	2.30 (2.00–3.50)	<0.001
Median age at surgery (range)	60 (27–80)	61 (31–83)	56 (27–78)	0.43
Median tumor volume before resection (cc) (range)	23.4 (1.1–170.6)	24.2 (2.3–90.1)	19.9 (1.1–170.6)	0.32
Median residual tumor volume after resection (cc) (range)	0.2 (0–12.1)	0.2 (0–5.9)	0.6 (0–12.1)	0.29
Median resection rate (%) (range)	99.9 (32.6–100)	99.2 (37.8–100)	98.0 (32.6–100)	0.48
Median KPS score at surgery (range)	80 (50–100)	80 (50–100)	80 (50–100)	0.87
No. of patients with methylated MGMT (%)	22 (50%)	12 (48%)	10 (53%)	0.34
Median interval from surgery to Met-PET in months (range)	13 (11–30)	13 (11–30)	13 (11–18)	0.34
Median follow-up period from Met-PET (days) (range)	568 (111–2375)	759 (111–2375)	399 (135–1521)	0.03
No. of cycles of adjuvant temozolomide (range)	13 (12–30)	13 (12–30)	13 (12–18)	0.39
No. of patients of recurrence/dissemination within one year after Met-PET (%)	18 (41%)	4 (16%)	14 (74%)	< 0.001
**No**. **of lesions of recurrence within one year after Met-PET**				
Local recurrence	11	2	9	0.003
Distant recurrence	8	2	6	0.04
**No**. **of patients with dissemination within one year after Met-PET**				
Subdural dissemination	1	1	0	0.38
CSF dissemination	1	0	1	0.26

Met-PET, ^11^C-methionine positron emission tomography; KPS, karnofsky performance status; MGMT, O^6^-methylguanin-DNA methyltransferase; CSF, cerebrospinal fluid.

### Methionine uptake and tumor progression after TMZ

**Figure 1:**
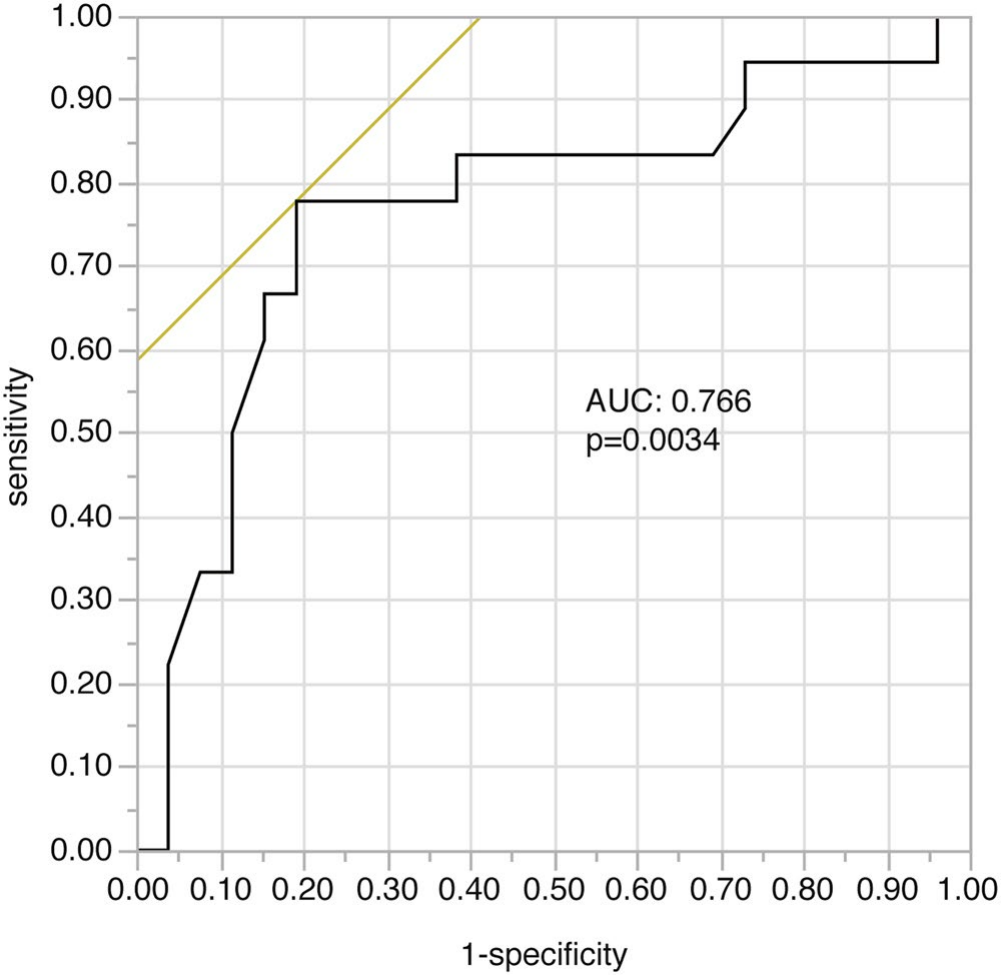
Receiver operating characteristic curve showing the status of tumor control at 12 months after Methionine PET in relation to methionine (Met) uptake. Met uptake significantly affected tumor control. AUC: area under the curve.

The ROC curve was evaluated to clarify the relationship between the Met uptake value after the extended TMZ treatment and recurrence within 12 months after Met-PET. The AUC was 0.766 (95% confidence interval [CI] 0.58–0.89, p = 0.003 (Fig. 1). A Tmax/Nave value of 2.0 was determined to be the optimal cut-off value for the best combination of sensitivity (77.8%) and specificity (80.8%). Patients with a Tmax/Nave value less than 2.0 (low uptake group) showed a risk reduction of 93% for any type of recurrence within one year after the PET scan (OR, 0.07; 95% CI, 0.02–0.30). Moreover, the low uptake group showed a 91% risk reduction for LR (OR, 0.09; 95% CI, 0.02–0.53). These findings suggested the strong predictive role of Met-PET in GBM patients who completed twelve or more cycles of extended TMZ treatment.

### Patient classification according the clarified cut-off Met-PET value

**Figure 2:**
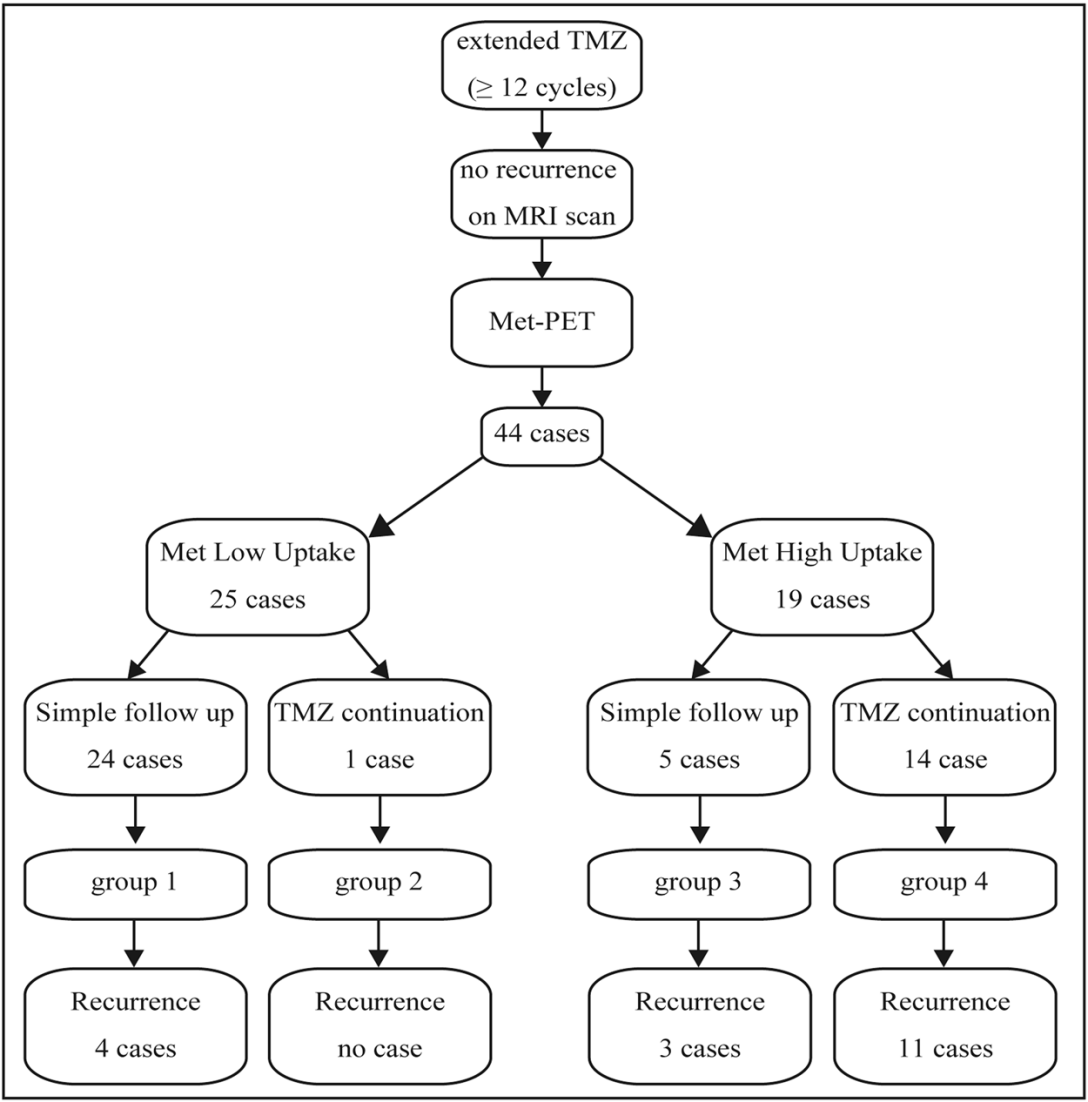
Clinical course of 44 patients who completed at least 12 cycles of adjuvant TMZ. The patients were retrospectively divided into two subgroups (low uptake and high uptake) according to the optimal Tmax/Nave cut-off value of 2.0 (See Fig. 1 and Results section for more detail). The 44 patients showed no evidence of tumor recurrence or progression on routine MRI surveillance. TMZ, temozolomide; MRI, magnetic resonance image; Met, methionine.

The 44 patients enrolled in this study were then divided into four subgroups based on a threshold Met-uptake value of 2.0 and the actual TMZ treatment strategy following Met-PET, which was TMZ treatment continuation or finalization with simple MRI follow-up (Fig. 2). As mentioned previously, patients with a Tmax/Nave value of 2.0 or more were classified into the high-uptake group (Table 1), which consisted of 19 patients, whereas the low-uptake group contained 25 patients. The median Met uptake value in the two subgroups was 2.30 (range; 2.00–3.50) and 1.56 (1.00–1.93), respectively. No significant difference in clinical background, including age, methylated MGMT promoter status, preoperative tumor volume, residual tumor volume after resection, and the resection rate was observed between the two groups. The duration of adjuvant temozolomide was not significantly different in the two groups (13 cycles in both groups).

### Choice of treatment after Met-PET

Figure 2 shows the clinical time course, the selected treatment, and detection of recurrence following Met-PET. A retrospective review of medical records revealed that the majority of patients (24 out of 25) in the low uptake group completed the extended TMZ treatment and were followed up with simple MRI surveillance monthly (group 1). Only one patient with a Met uptake value of 1.90 continued to receive TMZ even after Met-PET (group 2). In contrast, 14 out of 19 patients (74%) with a median uptake of 2.40 in the high-uptake group decided to continue the extended TMZ treatment (group 4). Five patients in the high-uptake group completed the extended TMZ treatment even without TMZ-related side effects and were followed up with monthly MRI (group 3). The median uptake value of Met was 2.16 in these 4 patients.

### The relationship between Met uptake and recurrence within one year after Met-PET

Figure 2 also demonstrates whether the tumor recurred or not within one year after the Met-PET examination. For the low Met uptake groups (groups 1 and 2), only four out of 25 patients (16%) who completed TMZ treatment experienced recurrence within one year after Met-PET, even though most of the low-uptake patients (24 out of 25) completed the TMZ treatment at the time of Met-PET (group 1). On the other hand, 14 out of the 19 (74%) patients in the high Met uptake group developed tumor recurrence within one year, showing that low Met uptake was associated with a 93% lower risk for recurrence within one year after the PET scan (OR, 0.07; 95% CI, 0.02–0.30). More specifically, three out of five patients who were followed up with simple MRI assessments and 11 out of 14 who continued the extended TMZ treatment showed recurrence.

### Illustrative case presentation

**Figure 3:**
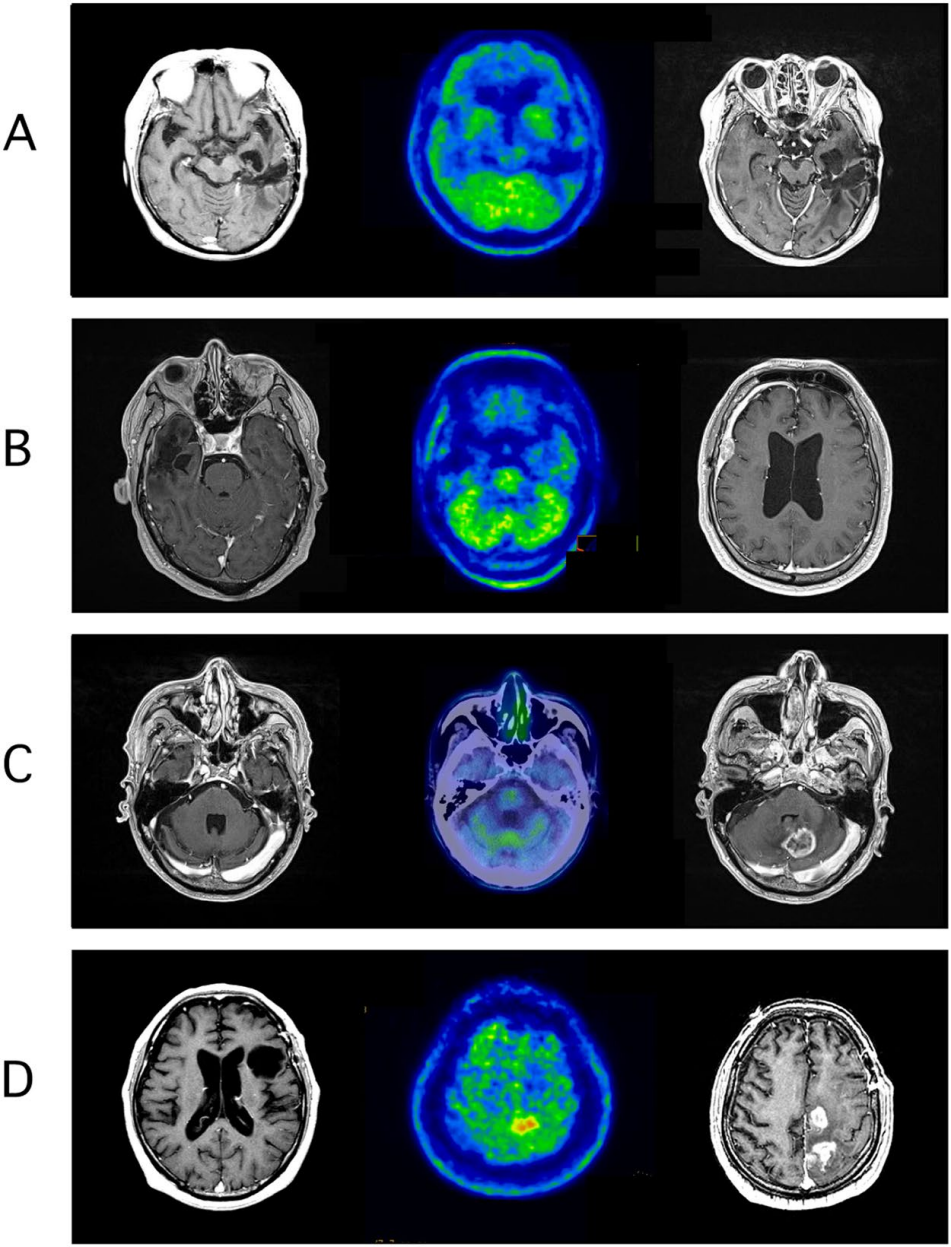
Illustrative presentations of four cases. Low methionine uptake (Tmax/Nave = 1.45) and no recurrence on finalizing the extended temozolomide (3A for group 1). Low methionine uptake (Tmax/Nave = 1.52) and finalization of the extended temozolomide followed by atypical subdural dissemination (3B for group 2). Low methionine uptake (Tmax/Nave = 1.48) and finalization of the extended temozolomide followed by local recurrence (3C for group 1). High methionine uptake (Tmax/Nave = 2.5) followed by distant recurrence even with continuation of the extended temozolomide (3D for group 4). For group classifications, see Fig. 2. For more details, see Results section.

Figure 3A demonstrates the typical images of a man in his 60’s in the low Met uptake group with left-temporal GBM. The tumor had an unmethylated MGMT promotor and the patient underwent conventional radiotherapy of 60 Gy plus concomitant TMZ. MRI (Fig. 3A, left) scan and Met-PET (Fig. 3A, middle) after 12 cycles of adjuvant TMZ treatment revealed complete disappearance of the tumor and a Tmax/Nave value of 1.45. Simple monthly MRI surveillance was offered (group 1), and the MRI scan (Fig. 3A, right) obtained 5 years after Met-PET confirmed no recurrence of the tumor. In another man in his 50’s with right temporal GBM with an unmethylated MGMT promotor, an MRI scan after 12 cycles of adjuvant TMZ treatment showed complete remission of the tumor (Fig. 3B, left), and Met-PET demonstrated low uptake (Tmax/Nave, 1.52) (Fig. 3B, middle). He also chose simple monthly MRI follow-up (group 1). Unfortunately, atypical subdural dissemination was histologically confirmed at 298 days after Met-PET (Fig. 3B, right). In Fig. 3C, a case with cerebellar GBM demonstrated LR after extended TMZ treatment (Fig. 3C, left) and confirmation of low Met uptake (Fig. 3C, middle) with a Tmax/Nave value of 1.48, suggesting low spatial resolution of PET. Another man in his 50’s with left frontal GBM with methylated MGMT promoter continued to receive extended TMZ treatment after confirmation of no recurrence/residue of the tumor with routine MRI (Fig. 3D, left) but with high Met uptake at the distant ipsilateral superior parietal lobule (Fig. 3D, middle) with a Tmax/Nave value of 2.50 (group 4). However, an MR image obtained 63 days after the Met-PET scan (Fig. 3D, right) showed evidence of recurrence at the same lesion as high Met uptake.

### Progression-free survival and pattern of recurrence

**Figure 4:**
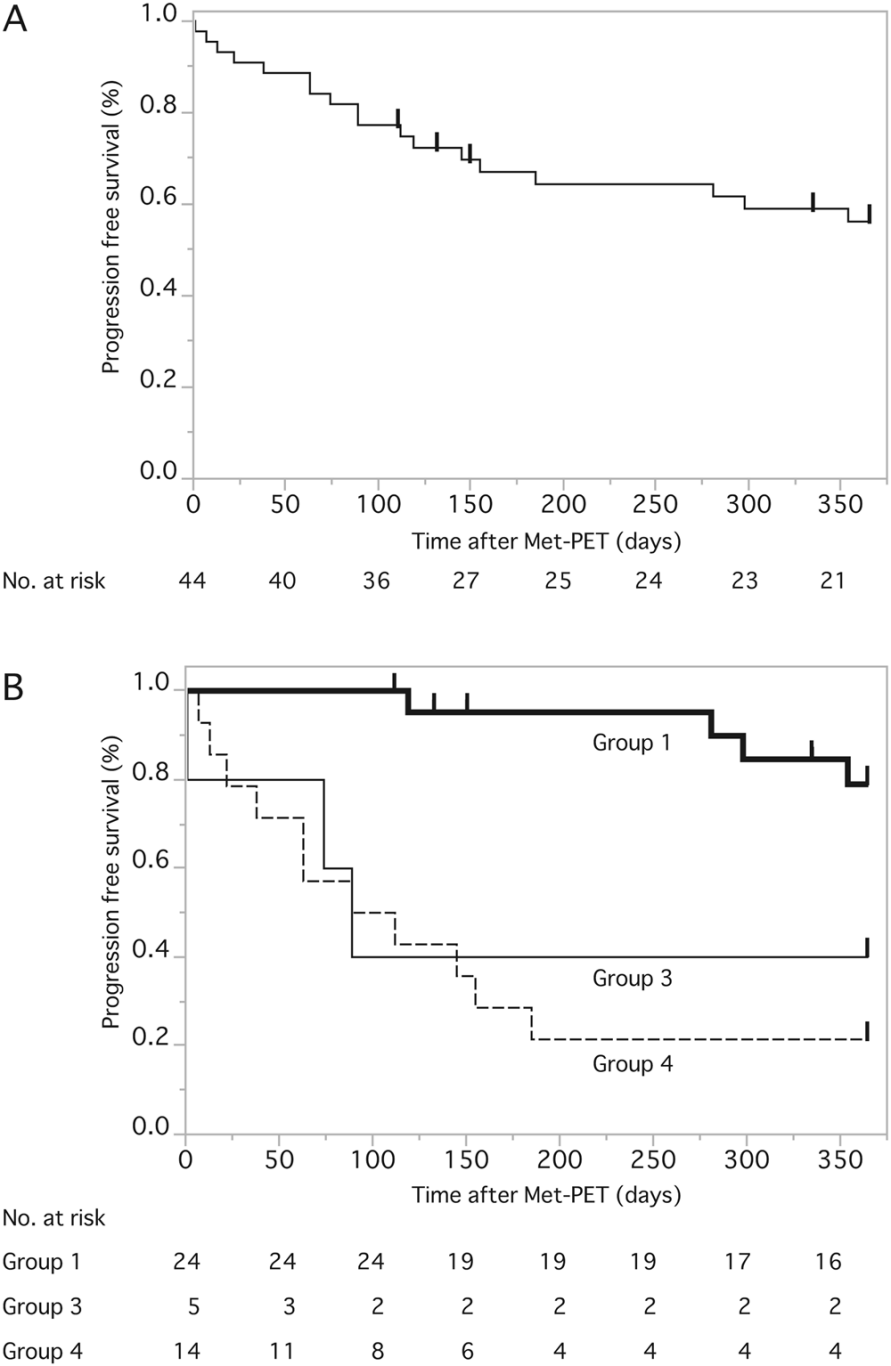
(**A**) A Kaplan–Meier curve for progression free survival (PFS) rate for 44 patients with a one-year PFS of 54.7% (95%CI, 39.0–69.4). (**B**) PFS rate in the subgroups was 78.0% (53.9–91.5), 40.0% (12.3–87.7) and 20.0% (6.6–47.0) (p < 0.0001, log-rank) according to methionine uptake status and temozolomide finalization or continuation. For more details, see Results section.

The Kaplan–Meier curve for PFS in all 44 patients after Met-PET is shown in Fig. 4A, with a one-year PFS of 54.7% (95% CI, 39.0–69.4). Next, to clarify the associations among the Met uptake value over at least 12 cycles of adjuvant TMZ therapy, treatment choices, and tumor progression (Fig. 2), PFS with patients divided into four subgroups was again calculated. For group 2, the case showing low uptake followed by TMZ treatment continuation was excluded from the detailed PFS analysis because of the small number of cases (n = 1). The PFS rate at one year after Met-PET in the remaining three subgroups was 78.0% (95% CI, 53.9–91.5), 50.0% (12.3–87.7), and 20.0% (6.6–47.0) (p < 0.0001, log-rank) respectively (Fig. 4B). These data clearly demonstrate that the recurrence rate in group 1 was very low (17%) but very high in group 3 (64%) and group 4 (79%) in a stepwise manner according to the median Met uptake value (1.55 in group 1, 2.20 in group 3, and 2.35 in group 4), and also showed more frequent tumor progression than patients with low uptake (<2.0) who completed the extended temozolomide treatment (p < 0.001, odds ratio 14.7, 95% CI 3.46–62.3).

The pattern of recurrence was also documented according to the original tumor location, Met uptake site, and location of recurrence. In group 1, 4 patients showed five recurrent tumors, including 2 LR, 2 DR, and one subdural dissemination. The patient in group 2 did not develop any type of recurrence. In group 3, one case each of LR, DR, and CSF dissemination were observed and eight cases of LR and five of DR were observed in group 4. There were no obvious trends in the pattern of recurrence and Met uptake.

## Discussion

The Stupp regimen remains the standard of care for patients with newly diagnosed GBM with feasible tolerability and acceptable toxicity^1,2,18^, but it shows far from satisfactory outcomes. Various modifications in the Stupp regimen have been attempted for improvement of OS/PFS over the conventional six cycles of adjuvant TMZ treatment. One of them included the dose-dense TMZ to deplete MGMT in tumor cells by continuous daily TMZ treatment^19^, which failed to demonstrate improved efficacy in a phase III clinical trial^20^. Another approach was to extend the cycle of adjuvant TMZ treatment^8,21–24^. Hau *et al*. reported that prolonged adjuvant TMZ treatment over a median number of 13 cycles resulted in a median PFS of 14 months. However, more than 40% of their patients had grade III glioma^21^. Another study by Roldan demonstrated the survival benefit of extended adjuvant TMZ treatment (median, 11 cycles) of a longer OS of 24.6 months compared to the standard six cycles of adjuvant TMZ treatment^22^. Malkoun *et al*. conducted similar retrospective analyses for prolonged TMZ administration with detailed toxicity reports^23^. One-third of their patients with prolonged TMZ treatment (median, 6 cycles; range, 0–32) experienced grade 3 or greater hematological toxicities. Barbagallo^8^ revealed a positive correlation between the number of TMZ cycles and OS/PFS. According to their study, the median PFS/OS in prolonged TMZ (more than six cycles) and standard TMZ (up to six cycles) treatment were 28/8 months and 20/4 months, respectively. These data led to the recent clinical trials into extended (up to 12 cycles) adjuvant TMZ treatment^4,6,7,25–31^. Additionally, pooled analysis from four randomized prospective trials for newly diagnosed GBM demonstrated the slightly longer PFS in patients with prolonged TMZ treatment, especially in patients with methylated MGMT^10^. However, the prolonged TMZ strategy includes the possible risk of toxicity, cost-effectiveness, and secondary tumorigenesis. Therefore, in GBM patients with no evidence of tumor recurrence on MRI who continue to receive prolonged adjuvant TMZ treatment, it is strongly desirable to differentiate the subpopulations in whom the extended use of TMZ could be withhold.

To our knowledge, this is the first study involving GBM patients receiving extended adjuvant TMZ treatment with no evidence of tumor recurrence on MRI clarifying the relationships among Met uptake value, treatment strategy, and tumor progression. We clearly demonstrated in Fig. 1 that the Met uptake value at completion of 12 cycles or more of adjuvant TMZ treatment were significantly associated with tumor recurrence within the following 12 months. The optimal threshold Tmax/Nave value was calculated to be 2.0. According to this analysis, we retrospectively classified 44 patients into four subgroups (Fig. 2) and revealed that more than 80% of patients with low Met uptake achieved no tumor recurrence without additional administration of TMZ and simple MRI follow-up. These findings would have great advantage in the selection of patients who are candidates for finalization of extended TMZ, possibly avoiding TMZ-related toxicity, ensuring cost-effectiveness, and preventing secondary carcinogenesis. However, four cases showed recurrence despite the low Met uptake. However, four cases showed recurrence despite the low Met uptake; in these group 1 patients, it is possible that TMZ continuation after low uptake PET may delay or even prevent the tumor recurrence, however, the increased risk of side effects and secondary tumorigenesis must be considered.

Among 4 cases with recurrence in group 1, two showed LRs and two showed DRs and additional atypical subdural dissemination. The possible explanations for LR with low Met uptake (for example, Fig. 3C) are the fundamental limitations of low spatial resolution in PET. However, GBM can recur within a short interval and even a PET scanner with higher resolution and sensitivity may not enable the detection of an early sign of recurrence. Another hypothesis for atypical subdural dissemination is the dramatic phenotypic change induced by long-term TMZ use^11,32^. Rapp *et al*.^33^ assessed the recurrence pattern in GBM and observed LR, DR, and the combined pattern in 79%, 10%, and 10% of the patients, respectively. In our series of 44 patients, the incidence of LR and DR was almost same (11 LRs and 8 DRs), supporting the possible phenotype change induced by prolonged TMZ administration.

On the other hand, among the 19 patients with high Met uptake, 14 (74%) experienced tumor recurrence after extended TMZ treatment and Met-PET with a median Tmax/Nave value of 2.30. The recurrence pattern in this population showed 9 LRs, 6 DRs, and one CSF dissemination. This demonstrates the earlier and better sensitivity of Met-PET for predicting tumor recurrence compared to MRI because at the timing of the PET scan, no obvious recurrence was detected by MRI scans in all the patients. In addition, this trend was not affected by the chosen treatment strategy following high Met uptake, indicating the need to change the treatment options including bevacizumab^3,4^, tumor-treatment field^34,35^ with a non-invasive device emitting low-intensity, intermediate frequency electric fields.

In our current study, we demonstrated the following new roles of Met-PET in the management of newly diagnosed TMZ patients. One is related to better patient selection for finalizing long-term adjuvant TMZ use, and the other involves improved sensitivity to predict the tumor recurrence well in advance. However, this study has several limitations. The relatively small number of patients (n = 44) requires accumulation of data for further validation of the usefulness of Met-PET in adjuvant TMZ treatment. Additionally, the low spatial resolution of PET may also limit the accurate delineation of the uptake value and the location. Furthermore, not all GBMs shows contrast enhancement on MR images and GBM tumor cells exist in non-enhancing area. In this study, we only analyzed the contrast enhancing area as tumor volume, which may influence the result. Finally, these data were collected retrospectively, which necessitate a prospective study to confirm the result. Moreover, the optimal timing of Met-PET scan could not be clarified because most of patients underwent PET scan after the completion of 13 cycles of extended TMZ (Table 1). Earlier PET scan (such as soon after 6 cycles of TMZ) may be an option for future study. However, our data clearly demonstrate the new role of Met-PET in GBM patients who undergo the extended adjuvant TMZ therapy.

## Conclusion

Finalization of the extended TMZ treatment based on low Met-PET uptake value was demonstrated to be feasible with a low tumor recurrence rate. Met-PET can be used for predicting tumor progression in long-term GBM patients without evidence of recurrence on routine MRI scan.

## References

[1] Hegi ME (2005). MGMT gene silencing and benefit from temozolomide in glioblastoma. N Engl J Med.

[2] Stupp R (2005). Radiotherapy plus concomitant and adjuvant temozolomide for glioblastoma. N Engl J Med.

[3] Chinot OL (2014). Bevacizumab plus radiotherapy-temozolomide for newly diagnosed glioblastoma. N Engl J Med.

[4] Gilbert MR (2014). A randomized trial of bevacizumab for newly diagnosed glioblastoma. N Engl J Med.

[5] Stupp R (2014). Cilengitide combined with standard treatment for patients with newly diagnosed glioblastoma with methylated MGMT promoter (CENTRIC EORTC 26071-22072 study): a multicentre, randomised, open-label, phase 3 trial. Lancet Oncol.

[6] Perry JR (2017). Short-Course Radiation plus Temozolomide in Elderly Patients with Glioblastoma. N Engl J Med.

[7] Weller M (2017). Rindopepimut with temozolomide for patients with newly diagnosed, EGFRvIII-expressing glioblastoma (ACT IV): a randomised, double-blind, international phase 3 trial. Lancet Oncol.

[8] Barbagallo GM (2014). Long-term therapy with temozolomide is a feasible option for newly diagnosed glioblastoma: a single-institution experience with as many as 101 temozolomide cycles. Neurosurg Focus.

[9] Seiz M (2010). Long-term adjuvant administration of temozolomide in patients with glioblastoma multiforme: experience of a single institution. J Cancer Res Clin Oncol.

[10] Blumenthal DT (2017). Is more better? The impact of extended adjuvant temozolomide in newly diagnosed glioblastoma: a secondary analysis of EORTC and NRG Oncology/RTOG. Neuro Oncol.

[11] Johnson BE (2014). Mutational analysis reveals the origin and therapy-driven evolution of recurrent glioma. Science.

[12] Jager PL (2001). Radiolabeled amino acids: basic aspects and clinical applications in oncology. Journal of nuclear medicine: official publication, Society of Nuclear Medicine.

[13] Shinozaki N (2011). Discrimination between low-grade oligodendrogliomas and diffuse astrocytoma with the aid of 11C-methionine positron emission tomography. J Neurosurg.

[14] Terakawa Y (2008). Diagnostic accuracy of 11C-methionine PET for differentiation of recurrent brain tumors from radiation necrosis after radiotherapy. Journal of nuclear medicine: official publication, Society of Nuclear Medicine.

[15] Tanaka Y (2009). Glioma surgery using a multimodal navigation system with integrated metabolic images. J Neurosurg.

[16] Iuchi T (2015). Methionine Uptake and Required Radiation Dose to Control Glioblastoma. Int J Radiat Oncol Biol Phys.

[17] Wen PY (2010). Updated response assessment criteria for high-grade gliomas: response assessment in neuro-oncology working group. J Clin Oncol.

[18] Stupp R (2009). Effects of radiotherapy with concomitant and adjuvant temozolomide versus radiotherapy alone on survival in glioblastoma in a randomised phase III study: 5-year analysis of the EORTC-NCIC trial. Lancet Oncol.

[19] Hegi ME (2008). Correlation of O6-methylguanine methyltransferase (MGMT) promoter methylation with clinical outcomes in glioblastoma and clinical strategies to modulate MGMT activity. J Clin Oncol.

[20] Gilbert MR (2013). Dose-dense temozolomide for newly diagnosed glioblastoma: a randomized phase III clinical trial. J Clin Oncol.

[21] Hau P (2007). Safety and feasibility of long-term temozolomide treatment in patients with high-grade glioma. Neurology.

[22] Roldan Urgoiti GB, Singh AD, Easaw JC (2012). Extended adjuvant temozolomide for treatment of newly diagnosed glioblastoma multiforme. J Neurooncol.

[23] Malkoun N (2012). Prolonged temozolomide for treatment of glioblastoma: preliminary clinical results and prognostic value of p53 overexpression. J Neurooncol.

[24] Skardelly M (2017). Prolonged Temozolomide Maintenance Therapy in Newly Diagnosed Glioblastoma. Oncologist.

[25] Batich KA (2017). Long-term Survival in Glioblastoma with Cytomegalovirus pp65-Targeted Vaccination. Clin Cancer Res.

[26] Bloch O (2017). Autologous Heat Shock Protein Peptide Vaccination for Newly Diagnosed Glioblastoma: Impact of Peripheral PD-L1 Expression on Response to Therapy. Clin Cancer Res.

[27] Chinnaiyan P (2017). A randomized phase II study of everolimus in combination with chemoradiation in newly diagnosed glioblastoma: Results of NRG Oncology RTOG 0913. Neuro Oncol.

[28] Galanis Evanthia, Anderson S Keith, Miller C Ryan, Sarkaria Jann N, Jaeckle Kurt, Buckner Jan C, Ligon Keith L, Ballman Karla V, Moore Dennis F, Nebozhyn Michael, Loboda Andrey, Schiff David, Ahluwalia Manmeet Singh, Lee Eudocia Q, Gerstner Elizabeth R, Lesser Glenn J, Prados Michael, Grossman Stuart A, Cerhan Jane, Giannini Caterina, Wen Patrick Y (2017). Phase I/II trial of vorinostat combined with temozolomide and radiation therapy for newly diagnosed glioblastoma: results of Alliance N0874/ABTC 02. Neuro-Oncology.

[29] Nayak L (2017). Phase I trial of aflibercept (VEGF trap) with radiation therapy and concomitant and adjuvant temozolomide in patients with high-grade gliomas. J Neurooncol.

[30] Shenouda G (2017). A Phase 2 Trial of Neoadjuvant Temozolomide Followed by Hypofractionated Accelerated Radiation Therapy With Concurrent and Adjuvant Temozolomide for Patients With Glioblastoma. Int J Radiat Oncol Biol Phys.

[31] Yu A (2017). Report of safety of pulse dosing of lapatinib with temozolomide and radiation therapy for newly-diagnosed glioblastoma in a pilot phase II study. J Neurooncol.

[32] Stepanenko AA (2016). Temozolomide promotes genomic and phenotypic changes in glioblastoma cells. Cancer Cell Int.

[33] Rapp M (2017). Recurrence Pattern Analysis of Primary Glioblastoma. World Neurosurg.

[34] Stupp R (2017). Effect of Tumor-Treating Fields Plus Maintenance Temozolomide vs Maintenance Temozolomide Alone on Survival in Patients With Glioblastoma: A Randomized Clinical Trial. Jama.

[35] Rehman AA, Elmore KB, Mattei TA (2015). The effects of alternating electric fields in glioblastoma: current evidence on therapeutic mechanisms and clinical outcomes. Neurosurg Focus.

